# Comparison of Gut Microbiota and Metabolic Characteristics Between Miechongshu-Treated and Untreated Yili Horses

**DOI:** 10.3390/ani16071020

**Published:** 2026-03-26

**Authors:** Yuhui Ma, Jian Zhang, Xiaobin Li, Haili Zhao, Wenyuan Lu, Hai Li, Kailun Yang

**Affiliations:** 1 College of Animal Science, Xinjiang Agricultural University, Urumqi 830052, China; mayuhuim@126.com (Y.M.); 18723181520@163.com (J.Z.); lxb262819@163.com (X.L.); 2 Zhaosu County Bureau of Agriculture and Rural Affairs, Zhaosu County 835600, China; 15026032042@163.com (H.Z.); m17839750693@163.com (W.L.)

**Keywords:** Miechongshu, anthelmintic, Yili horses, gut microbiota, metabolic characteristics

## Abstract

Anthelmintic treatment is widely used in equine health management to control parasitic infections; however, its potential effects on the gut microbiota and metabolic characteristics remain poorly understood. In this study, twelve Yili horses were randomly assigned to two groups: a control group (C), which received no anthelmintic treatment, and a Miechongshu-treated group (T), which was orally administered Miechongshu at a dose of 0.36 g/kg body weight on day 0. Fecal samples were collected from the rectums of Yili horses on day 14 after administration, and differences in gut microbial composition and metabolic characteristics between the two groups were further examined using 16S rRNA gene sequencing in combination with LC–MS/MS-based metabolomic analysis. The results showed that there was no significant difference in α diversity (*p* > 0.05). At the phylum level, compared with Group C, the relative abundance of Halobacterota significantly increased (*p* < 0.05). At the family level, the relative abundance of Prevotellaceae in Group T was significantly higher than that in Group C, while the relative abundance of Christensenellaceae decreased (*p* < 0.05). At the genus level, the relative abundance of *Christensenellaceae_R-7_group* was significantly decreased (*p* < 0.05). A total of 98 differentially expressed metabolites were identified in the total ion mode. Among them, 64 were upregulated compared with Group C, and 34 were downregulated. KEGG analysis revealed that four enriched metabolic pathways showed significant differences. Metabolic pathways, tryptophan metabolism, purine metabolism, fatty acid biosynthesis and carotenoid biosynthesis differed between the two groups. Compared with Group C, the contents of the metabolites tetradecanoic acid and adenosine were significantly decreased, while tryptophol was significantly increased. These results indicate that anthelmintic treatment is associated with alterations in the intestinal microecological balance and metabolic homeostasis of the host, providing new insights for evaluating the use of anthelmintics and the management of intestinal health in horses.

## Simple Summary

Anthelmintic drugs are widely used to control intestinal parasite infections in horses; however, their potential effects on the gut microbiota and metabolic environment remain poorly understood. In this study, gut microbial composition and fecal metabolic characteristics were compared between Miechongshu-treated and untreated Yili horses using 16S rRNA gene sequencing and untargeted LC–MS/MS metabolomics. Differences in gut microbial communities were observed between the two groups, characterized by an increased abundance of Prevotellaceae and a decreased abundance of Christensenellaceae. Metabolomic analysis revealed distinct shifts in metabolic profiles between groups, with significant changes in pathways related to carbohydrate, lipid, purine, and tryptophan metabolism. Key metabolites such as tetradecanoic acid and adenosine were reduced, whereas tryptophol was increased in treated horses. These findings suggest that Miechongshu treatment is associated with remodeling of the gut microecosystem and metabolic functions, which may be related to improved intestinal health in Yili horses. This study provides new insights into the interactions among anthelmintic treatment, gut microbiota, and host metabolism, supporting more rational and health-oriented parasite control strategies in horses.

## 1. Introduction

The gut microbiota of the human or animal body is closely related to the health of the host. As an important “metabolic organ” of the host, the gut microbiota plays a crucial role in many physiological processes, such as the digestion and absorption of animal nutrients, energy metabolism, immune regulation and the maintenance of intestinal homeostasis [1]. Meanwhile, intestinal microorganisms can produce a diverse range of metabolic products, including short-chain fatty acids, amino acid-derived compounds, nucleosides, and indole derivatives produced through the fermentation of dietary carbohydrates, proteins and other nutritional substrates. These metabolites from microbial sources not only participate in the host’s energy and material metabolism but also affect intestinal barrier function, immune response and inflammatory response through the action of signal molecules, thus profoundly shaping the host’s health condition [2,3].

Parasitic diseases pose a significant threat to the health of equids. Gastrointestinal parasite infections, in particular, are prone to causing impaired growth and development, malnutrition, anemia, emaciation, diarrhea, gastroenteritis, and alternating episodes of constipation and diarrhea in horses. These infections also damage the gastrointestinal mucosa, leading to hemorrhage, and reduce the activity of certain gastrointestinal enzymes. In severe cases, they may result in intestinal obstruction, colic, and even life-threatening conditions [4,5]. Consequently, parasitic diseases have emerged as a persistent challenge that critically constrains the sustainable development of the equine industry. Moreover, there exist multidirectional and complex interactions among parasites, the intestinal microbiota, and the host, all of which exert mutual influences on one another [6]. During intestinal colonization, parasites alter the gut microbial community by sharing the intestinal microenvironment with the resident microbiota, thereby potentially inducing both physiological and pathological changes in the host. These alterations hold significant implications for animal health, nutrient metabolism, immune function, and disease development [7]. The colonization and proliferation of flagellated protozoa in the host small intestine can disrupt the ecological balance of the gastrointestinal commensal microbiota, leading to diarrhea in the host [8]. After infection with Entamoebiasis histolytica in the human body, the occurrence of diarrhea symptoms is related to the aggravation of parasitic invasion and an increase in *Prevotella copri* [9]. Helminth infection impairs the metabolic functions of the gut microbiota, leading to body weight loss and malnutrition in the host [10]. Therefore, maintaining gastrointestinal health and stability is crucial for preventing intestinal diseases and promoting overall host health. To effectively control parasite-induced damage, anthelmintic drugs are routinely and rationally administered in livestock production and wildlife conservation as a primary strategy for the prevention and control of parasitic infections [11]. While anthelmintic treatment directly targets intestinal parasites by inhibiting or eliminating them, it may also exert complex effects on the gut microbiota, thereby indirectly influencing the efficacy of antiparasitic therapy. Emerging evidence suggests that anthelmintic interventions can alter host susceptibility to parasitic infections through parasite–microbiota interactions, with gut microbiota remodeling contributing to the regulation of immunometabolic homeostasis and the enhancement of antiparasitic capacity [12,13]. Treatment with albendazole in individuals infected with hookworms leads to significant alterations in the composition and diversity of the gut microbiota in association with therapeutic responses [14]. Ivermectin treatment induces mild and transient alterations in gut microbial composition and metabolite production [15]. Mechanistically, ivermectin exerts its antiparasitic effects by modulating neurotransmission in parasites, primarily through activation of glutamate-gated chloride channels and γ-aminobutyric acid (GABA) receptors, leading to neuromuscular hyperpolarization and paralysis [16,17]. In contrast, albendazole, a benzimidazole anthelmintic, binds to parasite β-tubulin, thereby inhibiting microtubule polymerization, disrupting glucose uptake and energy metabolism, and ultimately resulting in parasite death [18,19,20]. Previous studies have highlighted that the gut microbiota represents a key determinant of treatment success when albendazole–ivermectin combination therapy is used to treat infections with flagellates and hookworms [21]. However, there are currently few studies exploring the potential impact of Miechongshu (a compound anthelmintic formulation containing albendazole and ivermectin) on the intestinal flora and metabolic characteristics of Yili horses. Therefore, the present study aimed to systematically characterize the alterations in gut microbial structure and composition, fecal metabolite profiles, and key metabolic pathways between Miechongshu-treated and untreated Yili horses by integrating 16S rRNA sequencing and LC–MS/MS-based untargeted metabolomics. The findings of this study provide fundamental theoretical insights and practical evidence for optimizing parasite control strategies and standardizing anthelmintic use in equine management.

## 2. Materials and Methods

### 2.1. Animal Ethics Statement

The experimental procedures described in this study were evaluated and approved by the Institutional Animal Care and Use Ethics Committee of Xinjiang Agricultural University, located in Urumqi, China (approval number: 2022020). All protocols adhered to the relevant ethical standards for animal research.

### 2.2. Anthelmintic Drug

Miechongshu (Batch No. 207526059; formulation: 0.36 g per tablet, each tablet containing 350 mg albendazole and 10 mg ivermectin) was purchased from Hanzhong Xintianyuan Animal Pharmaceutical Co., Ltd., Hanzhong, Shaanxi, China.

### 2.3. Experimental Animals

Twelve Yili horses, approximately 12 months of age with an average body weight of 202.42 ± 23.81 kg, were selected, comprising six males and six females. The horses were randomly allocated into two groups (*n* = 6 per group, with equal sex distribution), designated as Group C and Group T, for a 14-day deworming efficacy evaluation trial. Previous studies suggest that parasite clearance and initial stabilization of gut microbial communities generally occur within 1–2 weeks following anthelmintic treatment, making this time point suitable for evaluating treatment-associated changes [22,23]. Under identical feeding management and dietary nutritional conditions, the control group (Group C) received no deworming treatment, while horses in Group T were administered a single oral dose of Miechongshu at 0.36 g/kg body weight on day 0 of the trial. All horses were clinically healthy based on veterinary examination prior to the experiment, with no signs of systemic illness, diarrhea, or respiratory disease. Fecal examinations confirmed that all horses were naturally infected with gastrointestinal parasites at baseline, with no history of anthelmintic or antibiotic treatment in the three months preceding the study. Additionally, prior to deworming, fecal samples from all Yili horses were examined for the species and counts of parasite eggs. The average infection rates were similar between the two groups, with relatively high infection rates of *Capillaria* spp., *Triodontophorus* spp., and *Strongyloides westeri*. At 14 days post-deworming, Group C still exhibited relatively high infection rates of *Parascaris equorum*, *Strongylus* spp., *Capillaria* spp., and *Triodontophorus* spp., whereas no parasite eggs were detected in Group T [22]. Due to the limited availability of experimental animals, a total of 12 Yili horses were included in this study (n = 6 per group). Therefore, this study should be considered a preliminary exploratory study intended to provide initial information on potential associations between anthelmintic treatment status and gut microbiota composition and metabolic profiles in Yili horses.

### 2.4. Feeding Management

The experimental horses were housed individually in separate stalls. They were fed in the stalls from 8:00 AM to 12:00 PM and allowed to exercise in the paddock from 12:00 PM to 8:00 AM. During the experimental period, high-quality hay was offered three times daily at 10:00 AM, 2:00 PM, and 8:00 PM, with free access to food and water. The hay consisted primarily of mountain grass, with a nutritional composition of 90.13% dry matter (air-dried basis), 10.17% crude protein, and 22.62% crude fiber. The anthelmintic treatment was administered orally. To prevent cross-infection between the experimental and control groups, the horses from each group were housed and exercised in different stalls and paddocks, with a distance of at least 30 m between groups. The diet composition and nutrient levels are presented in Table S1.

### 2.5. Sample Collection

At 9:00 AM on day 14 of the experiment, prior to the morning feeding, rectal fecal samples were obtained from each horse using sterile disposable long-arm gloves. The collected material was transferred into 10 mL cryogenic tubes that were sterile and free of nucleases, followed by immediate snap-freezing in liquid nitrogen. All samples were then preserved at −80 °C until further analyses, including characterization of gut microbial communities and metabolomic profiling.

### 2.6. Sample Determination

#### 2.6.1. Fecal Microbial Community

Approximately 200 mg of rectal fecal material was used for genomic DNA isolation with a Stool DNA Kit (Omega Bio-tek, Norcross, GA, USA), following the supplier’s protocol. The V3–V4 regions of the bacterial 16S rRNA gene were subsequently amplified using the primer pairs 338F (5′-ACTCCTACGGGAGGCAGCA-3′) and 806R (5′-GGACTACHVGGGTWTCTAAT-3′). Amplified fragments were resolved on 2% agarose gels, excised, and purified employing an AxyPrep DNA Gel Extraction Kit (Axygen Biosciences, Union City, CA, USA). The resulting amplicons were quantified, pooled at equimolar ratios, and sequenced using the Illumina NovaSeq 6000 platform (Illumina Inc, San Diego, CA, USA) with a paired-end strategy. Sequence reads were clustered into operational taxonomic units (OTUs) at a 97% similarity cutoff using UPARSE (v11.0.667). Representative sequences from each OTU were taxonomically annotated with the RDP classifier at a confidence threshold of 70%. The composition and relative abundance of microbial communities across phylum, family, and genus levels were visualized using bar charts generated in R (v3.6.0). Alpha-diversity metrics, including Chao1, Shannon, Simpson, ACE, Coverage, and PD_whole_tree indices, were calculated via QIIME2 (version 2020.6) to assess within-sample diversity. Differences in community structure among samples (beta diversity) were examined using QIIME, (version 1.9.1) incorporating principal component analysis (PCA) and principal coordinate analysis (PCoA). Specifically, PCoA was conducted based on weighted UniFrac distance matrices at the OTU level, and the resulting patterns were displayed graphically. To reduce potential bias arising from uneven sequencing depth, all datasets were normalized through rarefaction prior to diversity analyses. Following this procedure, Good’s coverage averaged 99%, indicating that sequencing depth was sufficient to capture the majority of microbial diversity present in the samples. Differentially abundant taxa between experimental groups were identified using linear discriminant analysis effect size (LEfSe), with a threshold LDA score > 4. In addition, Tax4Fun (v0.3.1) was employed to infer functional profiles of the microbial communities and to explore potential variations in metabolic pathways across treatments.

#### 2.6.2. Fecal Metabolomics Analysis

Each fecal sample (~100 mg) was first cryogenically pulverized under liquid nitrogen. The obtained homogenized powder was then suspended in pre-cooled 80% methanol and vigorously mixed using a vortex mixer. Following this, the mixture was kept on ice for 5 min and subsequently centrifuged at 15,000× *g* for 20 min at 4 °C. An aliquot of the supernatant was collected and diluted with LC-MS-grade water to adjust the methanol concentration to 53%. The diluted extract was transferred into new Eppendorf tubes and subjected to a second centrifugation under the same conditions. Finally, the clarified supernatant was collected and directly used for LC-MS/MS analysis.

Metabolomic profiling was conducted using a Vanquish UHPLC platform (Thermo Fisher Scientific, Bremen, Germany) interfaced with an Orbitrap Q Exactive™ HF or Q Exactive™ HF-X mass spectrometer (Thermo Fisher Scientific, Bremen, Germany) at Novogene Co., Ltd. (Beijing, China). Chromatographic separation was achieved on a Hypersil Gold column (100 × 2.1 mm, 1.9 µm particle size) using a linear gradient elution over 12 min at a constant flow rate of 0.2 mL/min. The mobile phase system consisted of solvent A (water supplemented with 0.1% formic acid) and solvent B (methanol). The gradient elution program was configured as follows: 0–1.5 min, 2% B; 1.5–4.5 min, 2–85% B; 4.5–14.5 min, 85–100% B; 14.5–15 min, 100–2% B; followed by 15–17 min at 2% B to re-equilibrate the column. Mass spectrometric detection was performed in both positive and negative electrospray ionization modes. Instrument parameters were set as follows: spray voltage, 3.5 kV; capillary temperature, 320 °C; sheath gas pressure, 35 psi; auxiliary gas flow rate, 10 L/min; S-lens RF level, 60; and auxiliary gas heater temperature, 350 °C.

Raw UHPLC–MS/MS data were analyzed using Compound Discoverer 3.3 (CD 3.3, Thermo Fisher Scientific) to perform peak detection, alignment, and metabolite quantification. The principal processing parameters included calibration of peak areas based on the first quality control (QC) sample, a mass accuracy threshold of 5 ppm, a signal intensity deviation tolerance of 30%, and the application of a predefined minimum intensity cutoff. Subsequently, peak intensities were normalized against the total ion signal to reduce systematic variation. Using the normalized dataset, molecular formulae were inferred by integrating information from adduct ions, precursor ions, and fragment ion spectra. Metabolite identification was achieved through comparison with public and in-house databases, including mzCloud, mzVault, and MassList, enabling both qualitative annotation and relative quantification. Downstream statistical analyses were conducted using R (version 3.4.3), Python (version 2.7.6), and the CentOS 6.6 operating system. For variables that deviated from a normal distribution, a normalization approach was applied using the following calculation: the raw quantified value of each sample divided by the ratio of the total metabolite abundance in that sample to the total abundance in QC sample 1, thereby generating a relative peak area. Features exhibiting a coefficient of variation (CV) exceeding 30% in QC samples were removed to ensure data reliability. The remaining dataset was used to generate the final metabolite identification and relative quantification results.

### 2.7. Statistical Analysis

All statistical evaluations were conducted using Student’s *t*-test where appropriate. Prior to analysis, data distribution was examined using the Shapiro–Wilk test to assess normality, while homogeneity of variance was determined via Levene’s test. Only datasets meeting these assumptions were subjected to parametric testing. For comparisons involving multiple groups, analysis of variance (ANOVA) was employed, and when a significant effect was identified (*p* < 0.05), post hoc pairwise differences were further explored using Tukey’s Honestly Significant Difference (HSD) test. All computations were carried out in SPSS software (version 27, IBM, Armonk, NY, USA). To minimize the risk of type I errors associated with multiple testing, *p*-values were adjusted using the Benjamini–Hochberg false discovery rate (FDR) correction, with an adjusted *p* < 0.05 considered indicative of statistical significance.

Metabolite annotation was performed by comparing the identified compounds against multiple reference databases, including KEGG, HMDB, and LIPID Maps (all accessed on 19 October 2025). For multivariate analysis, metabolomic datasets were preprocessed and normalized using MetaX software (version 2.0), followed by partial least squares discriminant analysis (PLS-DA) to explore group separation patterns. In addition, univariate statistical testing was carried out using Student’s *t*-test to assess differences in individual metabolites between two groups. To account for the increased likelihood of false-positive findings due to multiple comparisons, *p*-values were adjusted using the Benjamini–Hochberg false discovery rate (FDR) method. Metabolites meeting the criteria of variable importance in projection (VIP) > 1, adjusted *p* < 0.05, and fold change (FC) ≥ 1.5 or ≤0.67 were considered significantly differential. Visualization of differential metabolites was conducted using volcano plots generated in R with the ggplot2 package (version 3.5.1), based on log_2_(FC) and −log_10_(*p*-value). Hierarchical clustering heatmaps were created using the Pheatmap package (version 1.0.12) after z-score standardization of metabolite abundances. Functional interpretation and pathway enrichment analyses were carried out using the KEGG database. Pathways were regarded as significantly enriched when the proportion of differential metabolites mapped to a given pathway exceeded the background expectation (x/n > y/N) and met a significance threshold of *p* < 0.05.

## 3. Results

### 3.1. Effects of Miechongshu on the Gut Microbiota of Yili Horses

#### 3.1.1. Venn Diagram Analysis Based on OTUs

**Figure 1:**
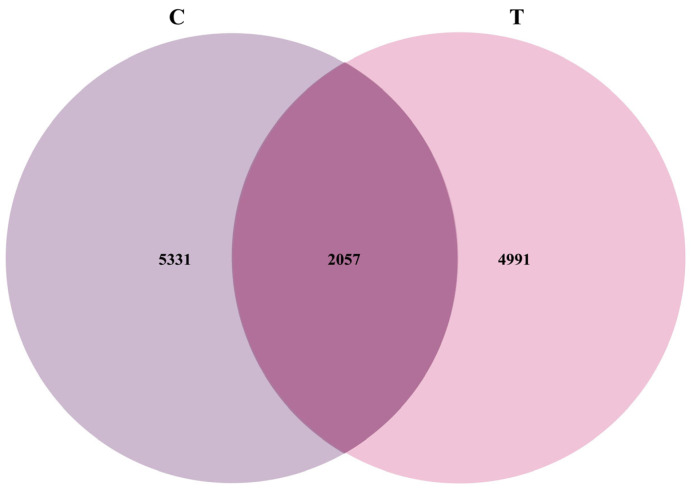
Venn diagram of OTUs.

As shown in Figure 1, the Venn diagram revealed that Group C and Group T shared 2057 OTUs, indicating a common species foundation and reflecting the basic similarity of the microbiota between the two groups. However, Group C contained 5331 unique OTUs, while Group T possessed 4991 unique OTUs, highlighting significant differences in microbial composition between the two groups.

#### 3.1.2. Analysis of Alpha Diversity

Alpha diversity represents both species richness and diversity within individual samples. The Good’s coverage values for both the C and T groups exceeded 99%, indicating that the sequencing depth was sufficient and that the fecal microbiota profiles were reliably captured. The results of the alpha-diversity assessment demonstrated that no significant differences were observed between Group C and Group T in terms of the Chao1, Shannon, Simpson, Pielou’s evenness, observed species, Faith’s PD, and Good’s coverage indices (Table 1). These findings suggest that treatment with the anthelmintic agent did not markedly influence the richness or diversity of the equine gut microbiota (*p* > 0.05).

#### 3.1.3. Beta-Diversity Analysis

**Figure 2:**
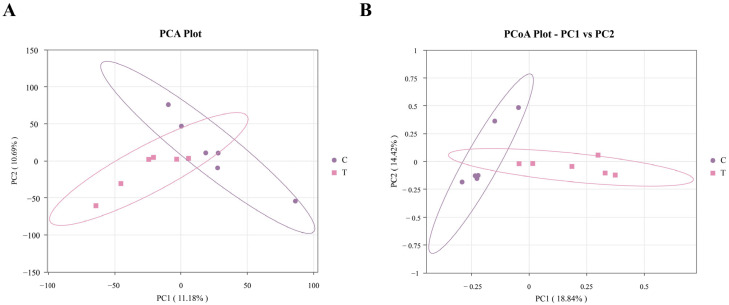
Principal component analysis (PCA) and principal coordinate analysis (PCoA). (**A**): PCA; (**B**): PCoA.

Principal component analysis (PCA) together with principal coordinate analysis (PCoA) was utilized to assess and visualize variations and similarities in microbial community structure between the control (C) and treatment (T) groups. As shown in Figure 2A, the first principal component (x-axis) explained 11.18% of the total variation, while the second principal component (y-axis) accounted for 10.69%, indicating a certain degree of separation in microbial composition between the two groups. PCoA-based β-diversity analysis provides a more accurate representation of the actual differences in community structure among samples, with the distance between sample points in the PCoA plot directly corresponding to the magnitude of dissimilarity calculated using the selected distance metrics. As shown in Figure 2B, the first principal coordinate (x-axis) accounted for 18.84% of the total variance, whereas the second coordinate (y-axis) contributed 14.42%. These results were consistent with those obtained from PCA, further suggesting that Miechongshu treatment exerted a modulatory effect on the gut microbiota of Yili horses.

#### 3.1.4. Structure and Relative Abundance of the Intestinal Microbiota

**Figure 3:**
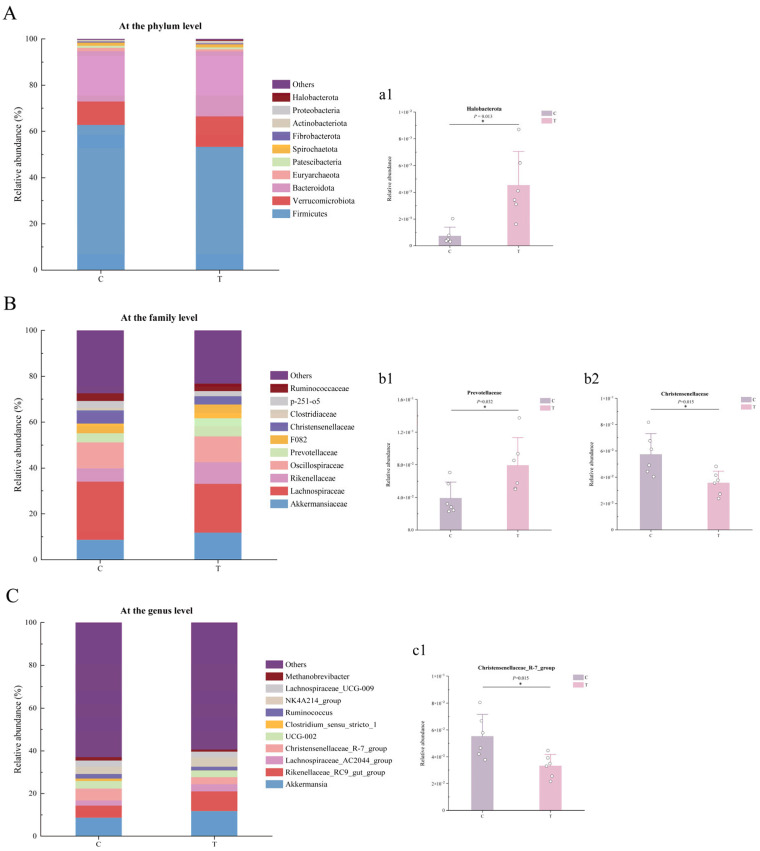
Relative abundance of intestinal microbiota at different taxonomic levels in Miechongshu-treated and untreated Yili horses. (**A**) Microbiota composition at the phylum level. The (**a1**) column plot indicates that the relative abundance of bacteria at the phylum level was significantly different between the C and T groups. (**B**) Microbiota composition at the family level. The (**b1**,**b2**) column plots indicate that the relative abundance of bacteria at the family level was significantly different between the C and T groups. (**C**) Microbiota composition at the genus level. The (**c1**) column plot indicates that the relative abundance of bacteria at the genus level was significantly different between the C and T groups. For (**a1**,**b1**,**b2**,**c1**), statistical significance was determined using *t*-tests. * means a significant difference (*p* < 0.05).

Based on PCA and PCoA, compositional differences in the intestinal microbiota between Group C and Group T were further compared. A more comprehensive assessment of microbial community composition in the two groups was performed by examining variations in relative abundance across multiple taxonomic levels (Figure 3) (Table S2). The relative abundances of the top 10 bacterial phyla are shown in Figure 3A, including Firmicutes, Verrucomicrobiota, Bacteroidota, Euryarchaeota, Patescibacteria, Spirochaetota, Fibrobacterota, Actinobacteriota, Proteobacteria, and Halobacterota. Among these, Firmicutes, Bacteroidota, and Verrucomicrobiota were the dominant phyla in both groups, accounting for more than 90% of the total relative abundance of the microbial community. The relative abundance of Halobacterota was significantly higher in Group T than in Group C (*p* = 0.013) (Figure 3(a1)).

Figure 3B presents the distribution of relative abundances of the top 10 bacterial families, including Akkermansiaceae, Lachnospiraceae, Rikenellaceae, Oscillospiraceae, Prevotellaceae, F082, Christensenellaceae, Clostridiaceae, p-251-o5, and Ruminococcaceae. Among these, Lachnospiraceae, Oscillospiraceae, Akkermansiaceae, and Rikenellaceae were the dominant families in both Group C and Group T. Compared with Group C, Group T exhibited a significantly increased relative abundance of Prevotellaceae, while the abundance of Christensenellaceae was markedly reduced (*p* = 0.032; *p* = 0.015) (Figure 3(b1,b2)).

Figure 3C shows the relative abundances of the top 10 genera, including Akkermansia, Rikenellaceae_RC9_gut_group, Lachnospiraceae_AC2044_group, Christensenellaceae_R-7_group, UCG-002, Clostridium_sensu_stricto_1, Ruminococcus, NK4A214_group, Lachnospiraceae_UCG-009, and Methanobrevibacter. In Group C, the dominant genera were Akkermansia, Rikenellaceae_RC9_gut_group, and Christensenellaceae_R-7_group. In Group T, the dominant genera were Akkermansia and Rikenellaceae_RC9_gut_group. Compared with Group C, the relative abundance of *Christensenellaceae_R-7_group* was significantly lower in Group T (*p* = 0.015) (Figure 3(c1)).

#### 3.1.5. Linear Discriminant Analysis (LDA) Coupled with Effect Size (LEfSe) Analysis and Functional Prediction of the Intestinal Microbiota Based on Tax4Fun

**Figure 4:**
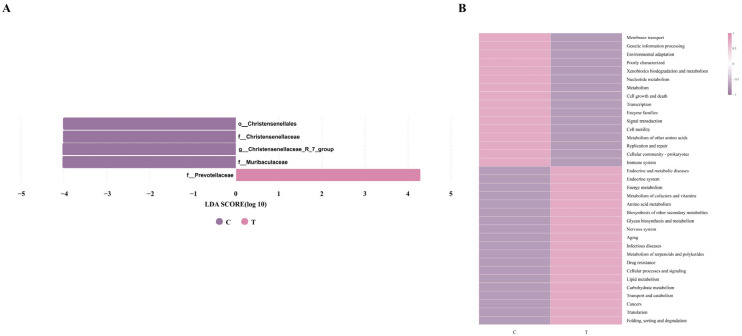
LEfSe analysis and functional prediction results of the intestinal microbiota. (**A**) LEfSe analysis identifying differential bacterial taxa (LDA score > 4, *p* < 0.05). (**B**) Tax4Fun functional clustering heatmap showing predictions of the intestinal microbiota; pink indicates high enrichment and purple indicates low enrichment.

To further elucidate the differences in the intestinal microbial communities between Group C and Group T, linear discriminant analysis (LDA) integrated with effect size estimation (LEfSe) was applied to detect taxa showing significant differences between the two groups across multiple taxonomic levels. As shown in Figure 4A, a total of five significantly differential taxa were identified at various taxonomic levels between Group C and Group T. Among these, Group C exhibited four significantly differential taxa: Christensenellales, Christensenellaceae, *Christensenellaceae_R-7_group*, and Muribaculaceae. In contrast, Group T displayed one significantly differential taxon, namely, Prevotellaceae. A total of 35 functional categories were predicted from the microbial community based on Tax4Fun. As shown in Figure 4B, Group T was positively correlated with 19 functional categories, including Endocrine and metabolic diseases, Endocrine system, Energy metabolism, Metabolism of cofactors and vitamins, Amino acid metabolism, Biosynthesis of other secondary metabolites, Glycan biosynthesis and metabolism, Nervous system, Aging, Infectious diseases, Metabolism of terpenoids and polyketides, Drug resistance, Cellular processes and signaling, Lipid metabolism, Carbohydrate metabolism, Transport and catabolism, Cancers, Translation, and Folding, sorting and degradation. In contrast, the functional prediction of the microbial community in Group C exhibited the opposite pattern to that observed in Group T.

### 3.2. Fecal Metabolic Profiles in Miechongshu-Treated and Untreated Yili Horses

#### 3.2.1. Correlation Analysis of QC Samples and Principal Component Analysis (PCA) in Untargeted Metabolomics

**Figure 5:**
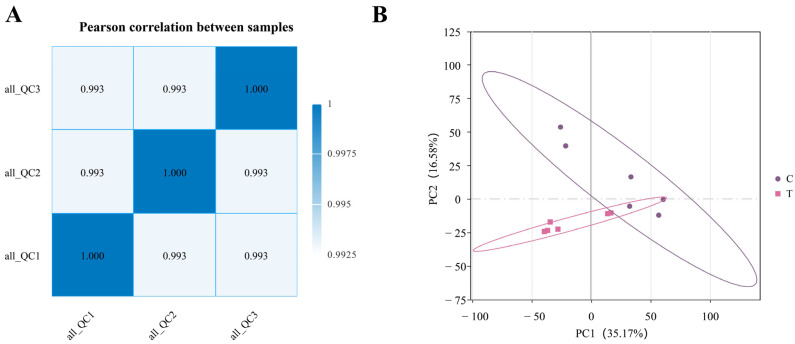
Correlation analysis and principal component analysis (PCA) of quality control (QC) samples. (**A**) Pairwise correlation assessment among QC samples. (**B**) PCA based on relative quantification of metabolites.

An untargeted metabolomics approach based on LC–MS/MS was utilized to comprehensively profile fecal metabolites of Yili horses in both groups. Quality control assessment was performed through correlation analysis of QC samples, serving as an essential indicator of data reliability. As presented in Figure 5A, correlation coefficients among QC samples, under the combined positive and negative ionization modes, all exceeded 0.99, with QC samples clustering closely together. Furthermore, PCA results (Figure 5B) indicated that the first principal component (PC1) and the second principal component (PC2) accounted for 35.17% and 16.58% of the total variance, respectively. Collectively, these findings demonstrate that the LC–MS/MS-based metabolomic data exhibited strong reproducibility and high analytical stability.

#### 3.2.2. Metabolite Classification and KEGG Pathway Annotation

**Figure 6:**
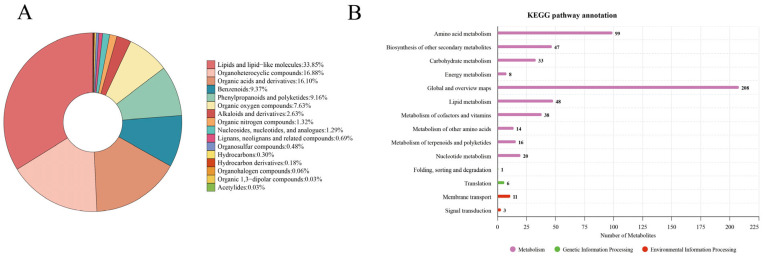
Metabolite classification and KEGG pathway annotation. (**A**) Overall metabolite classification pie chart. (**B**) KEGG classification annotation of the metabolome.

Results of statistical analysis of the chemical taxonomy of the identified metabolites and KEGG pathway annotation are shown in Figure 6. In total, 3341 structurally annotated metabolites were detected in this study (Table S3), which were classified into 16 categories. Among these, lipids and lipid-like molecules accounted for 33.85%, organoheterocyclic compounds for 16.88%, organic acids and derivatives for 16.10%, benzenoids for 9.37%, phenylpropanoids and polyketides for 9.16%, and organic oxygen compounds for 7.63%, and the remaining 7.01% were in other categories (Figure 6A). KEGG pathway analysis indicated that the most significantly enriched pathways were associated with global and overview maps, amino acid metabolism, and lipid metabolism (Figure 6B).

#### 3.2.3. Analysis of Differential Metabolites

**Figure 7:**
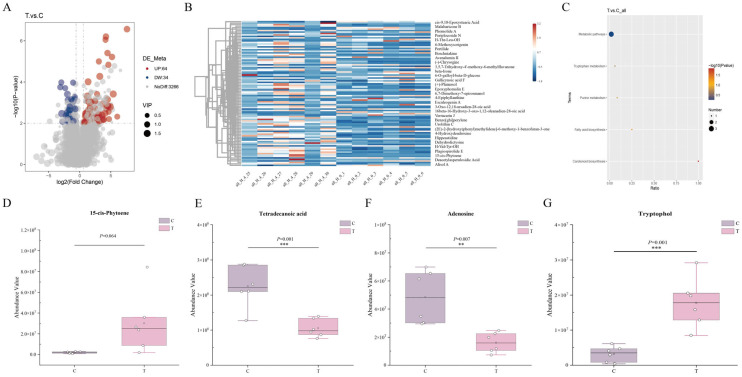
Analysis of differential metabolites. (**A**) Volcano plot of differentially expressed metabolites between the T vs. C groups. (**B**) Heatmap of differential metabolites. (**C**) Bubble plot of KEGG pathway enrichment of differential metabolites. (**D**–**G**) Key differential metabolites enriched in KEGG pathways: (**D**) 15-cis-phytoene; (**E**) tetradecanoic acid; (**F**) adenosine; (**G**) tryptophol. ** *p* < 0.01, and *** *p* < 0.001.

To identify differential metabolites between groups, the thresholds were set as VIP > 1.0, fold change (FC) ≥ 1.5 or ≤ 0.67, and *p* < 0.05 (Figure 7). PLS-DA analysis was performed to compare the metabolite profiles of Group C and Group T, revealing distinct separation between the two groups and indicating that deworming treatment induced significant alterations in the metabolic profiles of the horses. The validity of the PLS-DA model was further confirmed by permutation testing with 200 permutations, and the corresponding validation parameters (R^2^X, R^2^Y, and Q^2^) are presented in Figure S1. A volcano plot was generated to illustrate the distribution pattern of the identified differential metabolites (Figure 7A). The results showed that a total of 3364 metabolites were detected in Group C and Group T, of which 98 metabolites were significantly altered (64 upregulated and 34 downregulated). To intuitively display the expression levels of differential metabolites in each sample or group, a heatmap was generated to illustrate the relative abundance of these metabolites (Figure 7B). KEGG enrichment analysis of metabolic pathways revealed that metabolic pathways, tryptophan metabolism, purine metabolism, and fatty acid biosynthesis were significantly different between the two groups (Figure 7C). The carotenoid biosynthesis pathway showed a trend toward enrichment, in which treatment with Miechongshu tended to increase 15-cis-phytoene compared with Group C (*p* = 0.064) (Figure 7D); in the fatty acid biosynthesis pathway, tetradecanoic acid was significantly decreased (*p* = 0.001) (Figure 7E); in the purine metabolism pathway, adenosine was significantly decreased (*p* = 0.007) (Figure 7F); and in the tryptophan metabolism pathway, tryptophol was significantly increased (*p* = 0.001) (Figure 7G).

### 3.3. Correlation Analysis

**Figure 8:**
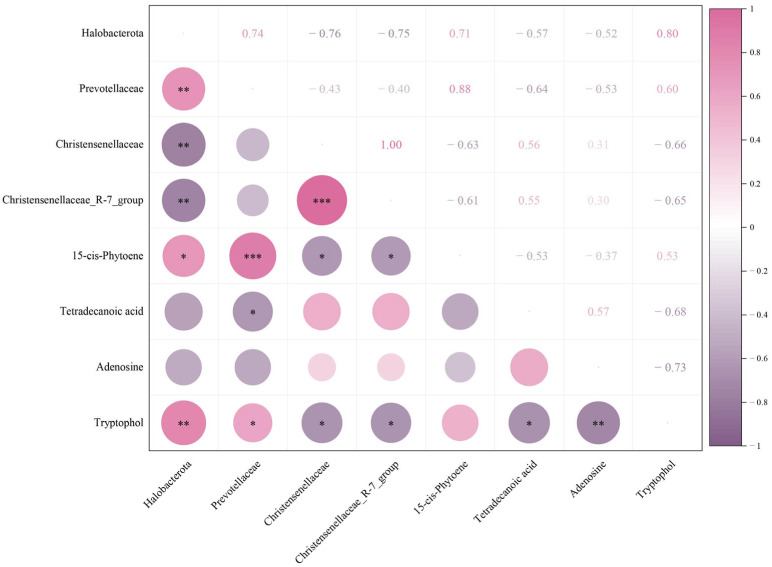
Correlation heatmap depicting associations between differential microbial taxa and key metabolites. The color scale represents the strength of Spearman’s correlation coefficients, with pink indicating positive correlations and purple indicating negative correlations. Asterisks denote statistical significance levels (* *p* < 0.05, ** *p* < 0.01, and *** *p* < 0.001).

To further elucidate the interactions between the gastrointestinal microbiota and metabolites in response to Miechongshu treatment, correlation analysis between differential microbiota and differential metabolites was performed using Spearman’s correlation coefficient (Figure 8). Halobacterota exhibited positive correlations with 15-cis-phytoene and tryptophol. Prevotellaceae was positively correlated with 15-cis-phytoene and tryptophol, but negatively correlated with tetradecanoic acid. Christensenellaceae and *Christensenellaceae_R-7_group* were negatively correlated with 15-cis-phytoene and tryptophol.

## 4. Discussion

The structure and composition of the gut microbiota in humans and animals can directly or indirectly participate in a wide range of host physiological functions, including nutrient digestion and absorption, intercellular signaling, resistance to pathogenic invasion, and regulation of the intestinal immune system [24]. At the same time, a variety of host-related factors, including genetic background, lifestyle, disease states and therapeutic interventions, may alter the composition and relative abundance of the gut microbiota [25]. Therefore, the gut microbiota and the host are interdependent and mutually influential, maintaining a symbiotic relationship shaped by co-evolution. However, animals are highly susceptible to parasitic infections during growth and development, and most parasitic infections exert detrimental effects on host health [26]. Not only are intestinal parasitic diseases major causes of diarrhea, nutritional disorders, anemia, and neurological dysfunction [27,28,29,30], but parasites residing within the host can also directly or indirectly disrupt intestinal homeostasis by modulating host immune responses and altering the structure of the gut microbiota [31], ultimately leading to impairment of this symbiotic relationship. The use of anthelmintics represents the primary and standard strategy for the prevention and control of parasitic diseases in livestock and poultry. On the one hand, anthelmintic treatment can directly and effectively suppress or eliminate intestinal parasites, thereby reducing parasite-induced mechanical damage to intestinal tissues and disruption of the intestinal microenvironment. On the other hand, anthelmintics may enhance resistance to parasitic infection at the mucosal level by modulating the composition of the gut microbiota and enhance the host’s systemic immune capacity against parasitic infections [32]. A previous study investigating the effects of anthelmintic treatment with Drontal Plus^®^ Tasty on dogs infected with *Toxocara canis* demonstrated that parasitic infection was associated with a reduction in gut microbial richness and diversity, whereas these indices increased following deworming. In addition, anthelmintic treatment markedly increased the relative abundance of *Bacteroidota* and *Segatella*, while reducing that of *Clostridium sensu stricto*. These alterations suggest that deworming may alleviate intestinal inflammation and metabolic dysfunction by promoting short-chain fatty acid production through modulation of the gut microbiota [33]. In a study investigating albendazole (400 mg) treatment for human hookworm infection, significant changes in gut microbiota composition were observed 10–14 days after a single dose of albendazole. Individuals who were successfully cured of hookworm infection exhibited a marked reduction in microbial diversity compared to their pre-treatment state, whereas those who failed to clear the infection showed no notable changes in microbiota composition. Notably, the microbiota composition and structure of successfully treated individuals were similar to those of uninfected participants [14]. In summary, anthelmintic treatments not only effectively control intestinal parasite infections but may also influence host gut microbiota. Despite their widespread use, research on the effects of anthelmintics on equine gut health remains limited. In this study, we investigated Yili horses to evaluate the impact of Miechongshu treatment on gut microbial composition and structure. Understanding these effects is crucial for guiding the rational application of anthelmintics and supporting the maintenance of gut health in the management of Yili horses.

Venn diagram and OTU-level α-diversity analyses indicated no statistically significant differences among groups, although numerical differences were observed. Specifically, the gut bacterial diversity indices of Group T were lower than those of Group C, suggesting that Miechongshu treatment moderately altered the diversity and richness of the gut microbiota in horses. Furthermore, β-diversity analysis revealed a clear separation in microbial community composition between the C and T groups, indicating that differences in microbial community structure were observed between the two groups on day 14 following treatment. Phylum-level analysis further revealed that Miechongshu treatment was associated with a higher relative abundance of Halobacterota in the equine gut microbiota. Previous studies have reported that members of Halobacterota are positively correlated with methane production and constitute an important archaeal group involved in methanogenesis [34,35,36]. The increased abundance of Halobacterota observed in this study may reflect alterations in the intestinal microbial environment following treatment. However, the mechanisms underlying this change remain unclear, and the functional roles of Halobacterota in the equine gut microbiome require further investigation.

In this study, anthelmintic treatment significantly increased the relative abundance of Prevotellaceae while decreasing that of Christensenellaceae. Genus-level analysis further revealed a significant reduction in the relative abundance of *Christensenellaceae_R-7_group*. Prevotellaceae are often reported to be relatively enriched under healthy dietary patterns and favorable metabolic conditions and have been associated with gut health and metabolic homeostasis. Members of this family have been reported to possess the ability to degrade complex carbohydrates (such as cellulose, hemicellulose, and pectin), including dietary fibers, resistant starches, and other polysaccharides that are otherwise indigestible by the host, leading to the production of short-chain fatty acids (SCFAs) such as acetate, propionate, and butyrate [37]. These metabolites may serve as important energy sources for intestinal epithelial cells and have been reported to participate in the regulation of host immune responses, including promoting the differentiation of anti-inflammatory Treg and Tr1 cells [38], modulating immune homeostasis, limiting the colonization of pathogenic bacteria, and contributing to the maintenance of intestinal barrier integrity [39]. *Christensenellaceae_R-7_group*, a member of the Christensenellaceae family, exhibits high heritability. Its abundance is significantly reduced in individuals with intestinal disorders or metabolic dysregulation and is considered a potential biomarker for maintaining gut health [40]. A study investigating the effects of different chemical drugs on the gut microbiota of naturally Eimeria-infected goats reported that Christensenellaceae, which promotes substrate degradation metabolism, exhibited a significant increase in relative abundance across treatment groups, whereas the relative abundances of Akkermansia and Bifidobacterium, genera associated with potential short-chain fatty acid production, were significantly reduced [41]. Differences between the findings of this study and those of previous research may be attributed to factors such as the experimental animals and the distinct metabolic pathways and pharmacological properties of different types of anthelmintics, which may consequently lead to differential regulatory effects on various microbial populations. Furthermore, Christensenellaceae and *Christensenellaceae_R-7_group* exhibited significant negative correlations with both 15-cis-phytoene and tryptophol, further reflecting the reorganization of the intestinal niche in the context of parasite clearance. The accumulation of these metabolites may be more closely associated with functionally dominant microbial populations that emerge following deworming, rather than with conventional homeostasis-associated microbiota. LEfSe analysis based on species differences revealed that the gut microbiota of horses in Group C was dominated by Christensenellaceae, whereas Miechongshu-treated horses in Group T were enriched with Prevotellaceae as the predominant differential taxa, consistent with the aforementioned results. Functional prediction based on Tax4Fun indicated that anthelmintic treatment primarily affected the gut microbial functions related to energy metabolism, amino acid metabolism, and carbohydrate metabolism in horses. The significant enrichment of carbohydrate metabolism functions indicates that anthelmintic treatment not only effectively clears intestinal parasites but also alters gut microbial composition. This shift may alleviate nutrient competition between parasites and the host, enhance the degradation of complex carbohydrates such as dietary fibers and non-starch polysaccharides, improve the efficiency of energy acquisition and conversion, and promote the restoration of host immunometabolic homeostasis, thereby indirectly influencing the physiological functions and overall health of Yili horses.

Gut metabolomics can systematically reflect the metabolic interactions between the gut microbiota and the host at the functional phenotypic level, serving as an important bridge linking changes in microbial community structure to host physiological effects [42,43]. Under conditions of nutritional intervention or drug treatment, the uptake and transformation of substrates by the gut microbiota, as well as the remodeling of metabolic networks, are often directly reflected in changes in metabolite composition and the enrichment patterns of metabolic pathways. Therefore, integrating bioinformatic analyses of untargeted metabolomics data can provide deeper insights into the potential effects of Miechongshu treatment on the host gut environment at the metabolite level. Untargeted metabolomics analysis based on LC-MS/MS revealed that, under the same feeding management and diet conditions, the fecal metabolic profiles of Yili horses were distinctly separated following Miechongshu treatment. The PLS-DA model demonstrated a clear distinction in metabolic phenotypes between the C and T groups, and permutation tests confirmed the model’s robustness and reliability. These results indicate that Miechongshu not only effectively clears intestinal parasites but also exerts a significant impact on the overall gut metabolic state. Regarding the overall metabolite composition, the metabolites identified in this study were classified into 16 categories, with the predominant category being lipids and lipid-like molecules, accounting for 33.85% of the total metabolites, followed by organoheterocyclic compounds at 16.88%. KEGG pathway enrichment analysis indicated that the identified metabolites were mainly associated with three major functional classes: metabolism, genetic information processing, and environmental information processing. Among these categories, metabolic pathways were the most prominently represented, highlighting their predominant role, within which 208 metabolites were associated with global and overview maps, and 99 metabolites were linked to amino acid metabolism. These findings align closely with the previously observed trends in microbiota functional prediction, particularly the significant changes in pathways related to energy metabolism, carbohydrate metabolism, and amino acid metabolism. This convergence suggests that Miechongshu may further influence metabolic profiles by reshaping the functional potential of the intestinal microbiota. Under the total ion mode, a total of 3364 differential metabolites were detected in the gut contents of horses between the C and T groups, of which 98 were significantly altered, including 64 upregulated and 34 downregulated metabolites. Further KEGG pathway enrichment analysis revealed that these differentially expressed metabolites were mainly enriched in four pathways: carotenoid biosynthesis (15-cis-phytoene), fatty acid biosynthesis (tetradecanoic acid), purine metabolism (adenosine), and tryptophan metabolism (tryptophol).

15-cis-Phytoene is an early key precursor in the carotenoid biosynthesis pathway and is widely present in plants, algae, fungi, and certain bacteria [44,45]. In the gut environment, carotenoids and their precursors function as antioxidants, protecting the intestinal epithelium and commensal microbes from potential oxidative damage by scavenging free radicals [46]. They are also involved in regulating intestinal immune homeostasis, as well as molecular expression processes and signal transduction pathways [47]. Mammals lack a complete endogenous pathway for carotenoid biosynthesis, and in the present study, feeding management and diet composition were consistent across all Yili horse groups. Previous studies have demonstrated that gut microbiota can participate in the transformation and accumulation of carotenoids and their precursor compounds [48]. In combination with the microbial community analysis in this study, it is therefore speculated that the significant enrichment of the carotenoid biosynthesis pathway may be associated with post-deworming ecological niche restructuring following parasite clearance, as well as alterations in gut microbial composition and metabolic function. Tetradecanoic acid, a medium-chain saturated fatty acid with broad-spectrum antimicrobial activity, has been shown to effectively inhibit the proliferation of various potential pathogenic bacteria, including *Escherichia coli* [49,50,51]. The antimicrobial activity of tetradecanoic acid is mainly associated with its capacity to inhibit the expression of the hemolysin gene (*hld*) in *Staphylococcus aureus*, as well as several genes related to biofilm formation in *Escherichia coli*, such as *csgAB*, *fimH*, and *flhD*. In addition, tetradecanoic acid has been shown to reduce cytotoxic effects in nematode infection models, thereby collectively contributing to the control of bacterial infections [52]. The results of the present study showed that Group T exhibited a significant reduction in the gut microbial metabolite tetradecanoic acid. This decrease reflects alterations in gut microbial metabolic activity or pathway utilization efficiency and suggests, to some extent, a suppression of metabolic processes associated with the synthesis or accumulation of saturated fatty acids within the intestinal environment. Adenosine is a key nucleoside in purine metabolism that regulates a wide range of physiological responses by activating specific receptors on the surface of cells or tissues. As an important mediator of energy conversion, adenosine plays a critical role in balancing energy production and consumption [53]. In addition, adenosine acts on adenosine receptors (A1, A2A, A2B, and A3) and functions as a signaling molecule involved in the regulation of intestinal immune responses, inflammatory status, and epithelial barrier function, thereby contributing to the maintenance of gut health [54,55,56]. In the present study, relatively higher levels of adenosine were detected in the gut of horses in Group C. This observation may be associated with the persistent presence of parasites, under which the host immune system may maintain immune tolerance or limit excessive inflammatory responses through adenosine-mediated signaling pathways. Following Miechongshu treatment, as parasite burden decreased, the intestinal immune environment may have undergone certain alterations, potentially reducing the requirement for adenosine-mediated immunoregulation and consequently resulting in a lower level of adenosine in the gut. Tryptophol is an important derivative produced through the metabolism and enzymatic conversion of tryptophan by specific gut microbial enzymes [57]. In many microbial communities, tryptophol functions as a quorum-sensing molecule involved in intercellular signaling and exhibits antimicrobial activity. In addition, tryptophol possesses antiviral and antitumor properties; for example, it has been reported to induce apoptosis in human leukemia U937 cells without affecting normal lymphocytes [58,59]. Following the clearance of parasites by Miechongshu treatment, intestinal ecological niches may undergo redistribution, potentially providing increased substrate availability or metabolic space for tryptophan-metabolizing microbial taxa, thereby enhancing the conversion efficiency of tryptophan to tryptophol. Correlation analysis revealed a significant positive association between Halobacterota and tryptophol. This finding suggests a potential relationship between this archaeal group and tryptophan-derived microbial metabolites. However, the present study only demonstrates an association, and the underlying mechanisms remain unclear. Further studies are required to determine whether Halobacterota directly or indirectly contributes to tryptophan metabolism in the equine gut. Collectively, these results offer further support for the inherent associations between gut microbial communities and their corresponding metabolites, indicating that Miechongshu may influence intestinal physiological functions and metabolic status by modulating key metabolites and metabolic pathways, ultimately affecting intestinal and overall host health. It is important to note that this study assessed the impact of Miechongshu administration within a relatively short observation window of 14 days. Therefore, the long-term impacts of this anthelmintic treatment on the intestinal microbiota and metabolic profiles of horses remain unclear. Future studies with extended observation periods are required to determine whether the microbial and metabolic changes observed in this study persist over time or represent transient responses following treatment.

## 5. Conclusions

In summary, this study demonstrates that Miechongshu treatment significantly modulates the gut microbiota and metabolic profiles of Yili horses. The observed shifts were characterized by an increased relative abundance of Prevotellaceae and a decreased abundance of Christensenellaceae, accompanied by significant alterations in key metabolic pathways, including reduced levels of tetradecanoic acid and adenosine and increased tryptophol. These findings suggest that, beyond its antiparasitic efficacy, Miechongshu induces remodeling of the host gut microecosystem, which may confer additional benefits to intestinal health. However, the absence of baseline measurements and the observational nature of this study preclude causal inferences, and the observed changes represent associations at day 14 post-treatment. Future longitudinal studies incorporating pre- and post-treatment sampling, multi-timepoint monitoring, metagenomic analysis, and host immune parameter assessments are warranted to elucidate the underlying mechanisms. Such investigations will provide a scientific basis for developing more precise and sustainable integrated strategies for parasite control in equine management.

## Figures and Tables

**Table 1:** Effect of Miechongshu on bacterial alpha diversity in the feces of Yili horses.

Item	C	T	*p*-Value
chao1	1845.9093 ± 194.4990	1764.3182 ± 247.8225	0.541
dominance	0.0118 ± 0.0192	0.0112 ± 0.0072	0.939
goods_coverage	0.9953 ± 0.0012	0.9968 ± 0.0016	0.099
observed_features	1765.3300 ± 203.9200	1734.1700 ± 243.7990	0.815
pielou_e	0.8335 ± 0.0655	0.8217 ± 0.0414	0.718
shannon	8.9912 ± 0.7975	8.8392 ± 0.5930	0.716
simpson	0.9882 ± 0.0192	0.9888 ± 0.0072	0.939

Note: Observed_features, Chao1 and Dominance indices were selected to identify community richness; Shannon and Simpson indices were used to identify community diversity; Good’s coverage index was used to calculate sequencing depth; Pielo_e index was used to calculate species evenness. Data are presented as mean ± standard deviation.

## Data Availability

The data that support the findings of this study are available from the corresponding author upon reasonable request.

## Supplementary Materials

The following supporting information can be downloaded at https://www.mdpi.com/article/10.3390/ani16071020/s1: Table S1: Ingredients and nutrient levels of basal diet (dry matter basis). Table S2: The relative abundance of intestinal microbiota in Miechongshu-treated and untreated Yili horses at the phylum, family, and genus levels; Table S3: Untargeted metabolomics analysis based on LC-MS/MS reveals the effects of Miechongshu on the intestinal metabolic profiles of Yili horses; Figure S1: PLS-DA score plot (A) and permutation test (B).
