# Supplementary material for: Comparison of Gut Microbiota and Metabolic Characteristics Between Miechongshu-Treated and Untreated Yili Horses
Source: Animals (Basel). 2026 Mar 26;16(7):1020. doi: 10.3390/ani16071020 (PMC13072335; doi:10.3390/ani16071020)
Supplement: Supplementary file 1 [file animals-16-01020-s001.zip › Table S1.pdf]

Table S1. Ingredients and nutrient levels of basal diet (dry matter basis).

Composition	Content (%)	Nutrient Index [2]	Nutritional Status
Mountain grass	78.75	OM (%)	92.15
Corn	11.58	CP (%)	15.50
Wheat bran	4.26	GE MJ/kg	27.32
Soybean meal	4.25	NDF (%)	35.15
Dicalcium phosphate	0.84	ADF (%)	27.37
NaCL	0.11	Ca (%)	0.75
Premix [1]	0.21	P (%)	0.36
Total	100		

[1] Premix provided the following per kilogram of diet: VA 480 IU; VB1 816.32 mg; VB2 333.2 mg; VB6 48.96 mg; VD 70.4 IU; VE 21,333.36 IU; pantothenic acid 20.46 mg; nicotinamide 484.85 mg; Cu 10.58 mg; Fe 35.56 mg; Mn 33.54 mg; Zn 30.92 mg; I 2.56; Se 5.93 mg; Co 1.11 mg. [2] Nutrient levels are measured values.
