# Supplementary figures and images for: Comparison of Gut Microbiota and Metabolic Characteristics Between Miechongshu-Treated and Untreated Yili Horses
Source: Animals (Basel). 2026 Mar 26;16(7):1020. doi: 10.3390/ani16071020 (PMC13072335; doi:10.3390/ani16071020)

**A**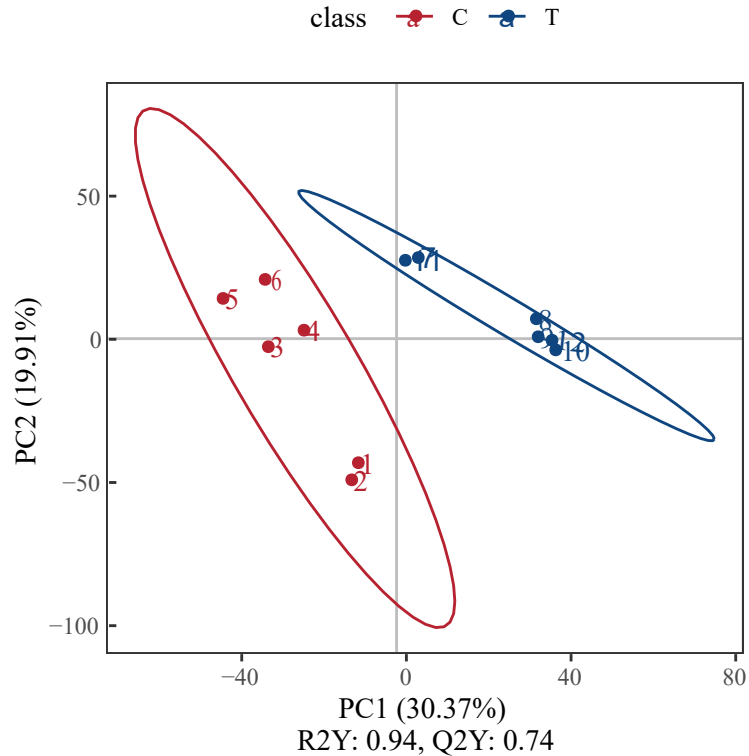**B**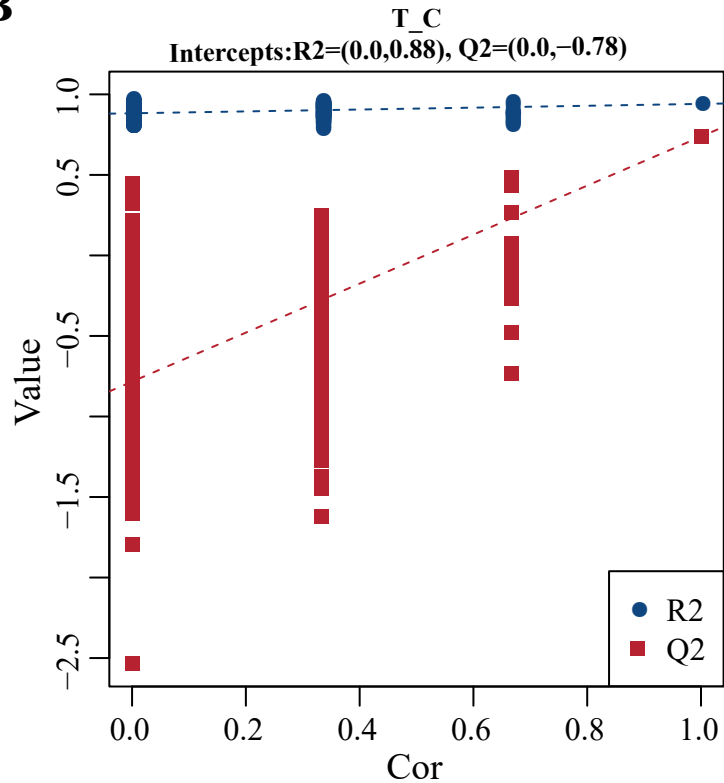

Supplement: Supplementary file 1 [file animals-16-01020-s001.zip › Figure S1.pdf]
